# Upon Accounting for the Impact of Isoenzyme Loss, Gene Deletion Costs Anticorrelate with Their Evolutionary Rates

**DOI:** 10.1371/journal.pone.0170164

**Published:** 2017-01-20

**Authors:** Christopher Jacobs, Luke Lambourne, Yu Xia, Daniel Segrè

**Affiliations:** 1 Bioinformatics Program, Boston University, Boston, Massachusetts, United States of America; 2 Department of Bioengineering, Faculty of Engineering, McGill University, Montreal, Quebec, Canada; 3 Department of Biology, Boston University, Boston, Massachusetts, United States of America; 4 Department of Biomedical Engineering, Boston University, Boston, Massachusetts, United States of America; CNR, ITALY

## Abstract

System-level metabolic network models enable the computation of growth and metabolic phenotypes from an organism’s genome. In particular, flux balance approaches have been used to estimate the contribution of individual metabolic genes to organismal fitness, offering the opportunity to test whether such contributions carry information about the evolutionary pressure on the corresponding genes. Previous failure to identify the expected negative correlation between such computed gene-loss cost and sequence-derived evolutionary rates in *Saccharomyces cerevisiae* has been ascribed to a real biological gap between a gene’s fitness contribution to an organism “here and now” and the same gene’s historical importance as evidenced by its accumulated mutations over millions of years of evolution. Here we show that this negative correlation does exist, and can be exposed by revisiting a broadly employed assumption of flux balance models. In particular, we introduce a new metric that we call “function-loss cost”, which estimates the cost of a gene loss event as the total potential functional impairment caused by that loss. This new metric displays significant negative correlation with evolutionary rate, across several thousand minimal environments. We demonstrate that the improvement gained using function-loss cost over gene-loss cost is explained by replacing the base assumption that isoenzymes provide unlimited capacity for backup with the assumption that isoenzymes are completely non-redundant. We further show that this change of the assumption regarding isoenzymes increases the recall of epistatic interactions predicted by the flux balance model at the cost of a reduction in the precision of the predictions. In addition to suggesting that the gene-to-reaction mapping in genome-scale flux balance models should be used with caution, our analysis provides new evidence that evolutionary gene importance captures much more than strict essentiality.

## Introduction

Quantitatively assessing the contribution of each gene to the overall fitness of an organism is an ongoing challenge in evolutionary and systems biology [1]. A classical, bioinformatics estimate of this contribution has been the evolutionary rate of the gene in question, which is based on genetic sequence conservation patterns amongst phylogenetically related genes [2–5]. This evolutionary rate metric serves as a historical record, providing a retrospective cumulative quantification of the importance of a gene. In contrast, systems biology methods are able to specifically quantify, for each gene, its current contribution to overall organism fitness by directly measuring [6,7] or estimating [8,9] the fitness defect caused by the removal of that gene. The natural question arises of whether the current contribution of a given gene to organism fitness, i.e. its dispensability, correlates with its historical importance. It is non-trivial whether such a relationship should exist, because the dispensability of any one gene at any set time point may be influenced by many complex factors, including the environmental condition(s) and its interactions with other genes within the genome, whose effects cannot be discerned from evolutionary rate. This question has been previously addressed in the model organism *Saccharomyces cerevisiae* (budding yeast) [10,11], for which fitness defect scores upon gene deletion have been experimentally measured in a systematic and comprehensive way [6,7,12,13]. Interestingly, a significant negative correlation between gene evolutionary rate and gene dispensability is detectable, although the signal is weak (Spearman’s *ρ* approx. −0.2).

In addition to the high-throughput experimental techniques used to quantify gene dispensability at the genome scale, constraint-based modeling techniques—such as flux balance analysis (FBA) [14]–may be used to efficiently generate such data *in silico* [15]. Flux balance models have been shown to successfully recapitulate several experimental observations, including growth phenotypes under various environmental conditions and gene essentiality in select lab conditions [16–18]. However, one of the puzzling failures of FBA techniques has been precisely the lack of even moderate correlation between predicted gene dispensability and evolutionary rate [11]. This lack of correlation has been ascribed to a number of possible reasons, including lack of knowledge about the most relevant environmental conditions to be used in simulations, and the complex condition-dependence of gene essentiality [8,11,13].

**Fig 1:**
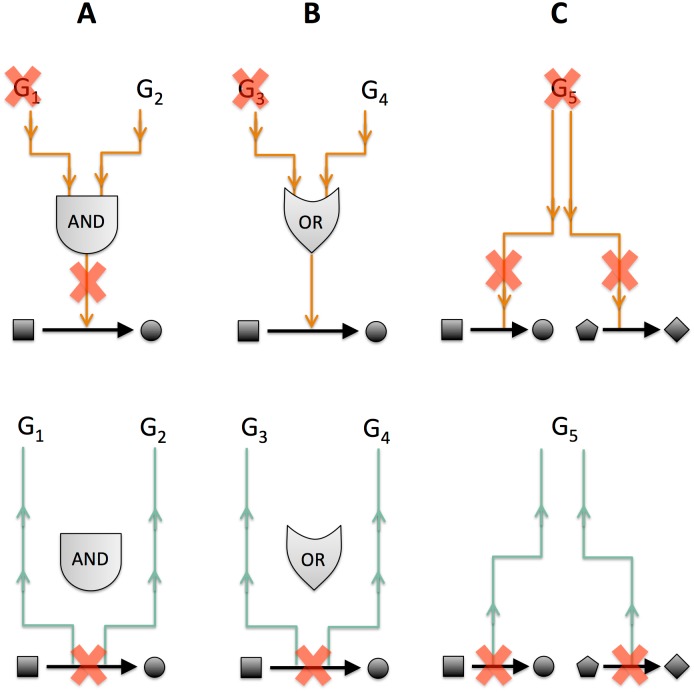
Comparison of the gene dispensability metrics: function-loss cost and gene-loss cost. Each toy scenario (A–C) represents a possible gene-to-reaction mapping configuration in its simplest form. Gene-loss cost (orange arrows, top row) propagates gene deletions “downwards” through logic gates to determine which reaction(s) are removed from the network, which in turn determine model fitness predictions. Function-loss cost (green arrows, bottom row) conceptually reverses this process, first calculating the fitness cost of removing each reaction in the network and then propagating these costs “upwards” to all associated genes, whereby they are summed together. For enzyme complexes (A), gene-loss cost and function-loss cost are identical and are equal to the fitness cost of the associated reaction's removal. For isoenzymes (B), the gene-loss cost is zero in all cases (because either gene will satisfy the logic gate's requirement that at least one enzyme is present), however the function-loss cost is as in scenario (A). For multi-function enzymes (C), the gene-loss cost is determined by the cost of removal of all reactions that are dependent on that gene according to the gene-to-reaction mapping, while function-loss cost is equal to the total summed cost of all its associated reactions' removal cost.

**Fig 2:**
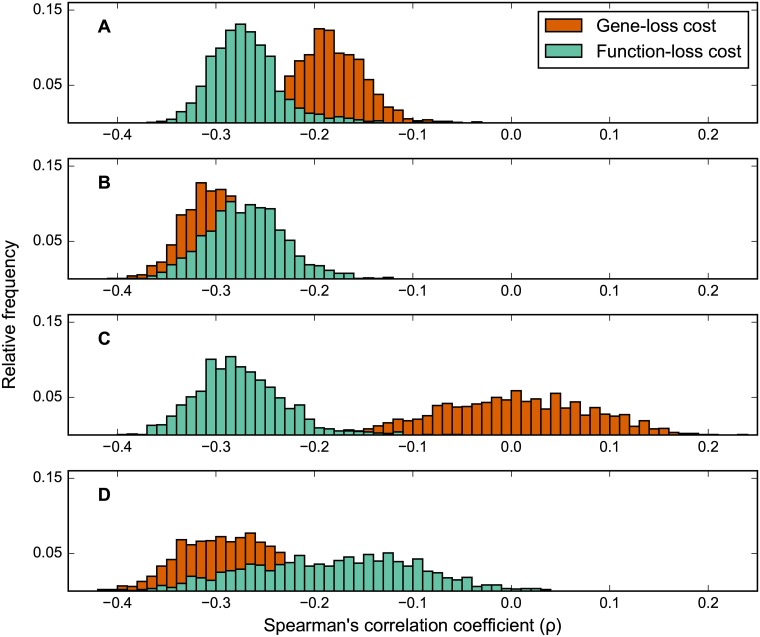
Frequency distributions of (Spearman’s rank) correlation between gene evolutionary rate and gene deletion impact scores. Frequency distributions of gene dispensability and evolutionary rate correlations. Gene evolutionary rate was calculated per gene as the average ranked *d*_N_/*d*_S_ (*K*_a_/*K*_s_) ratio between the *S*. *cerevisiae* gene and its ortholog in five related yeast species. A gene's deletion impact score was predicted with FBA using gene-loss cost (dark orange distributions) and function-loss cost (light green distributions). Rows show distributions for: (A) all genes, (B) all genes except isoenzymes, (C) isoenzymes only, and (D) only multi-functional enzymes that are **not** isoenzymes.

Here we present an alternative metric for measuring gene dispensability using FBA, which we call “function-loss cost” (Fig 1, light green arrows). As opposed to the standard “gene-loss cost” (Fig 1, dark orange arrows), our new metric estimates the total cost of a gene’s deletion by integrating the fitness costs of removing each enzymatic function associated with that gene from the FBA model, even if alternative isoenzymes exist for a given reaction. This is in contrast to the standard assumption in FBA models that isoenzymes associated with the same reaction act as completely redundant backups of each other. Using function-loss cost as our measure of gene dispensability, we are able to observe a negative correlation between the impact of gene deletion and gene evolutionary rate, that is significantly stronger than the same correlation calculated using gene-loss cost (Fig 2).

Furthermore, we find that our new treatment of isoenzymes in the model can also be informative in the study of genetic interactions, or epistasis [19]. The ability of FBA to predict the experimentally observed epistatic interaction [20] between any two metabolic gene deletions is changed when treating isoenzymes in this new way, with an increase in the true positive rate but also an increase in the false positive rate. Thus, function-loss cost provides novel insight about enzyme gene dispensability, while simultaneously suggesting that some standard assumptions used in genome scale modeling may not be universally applicable.

## Results

### Gene-loss cost and evolutionary rate correlate weakly in minimal environments

In prior work, it was established that gene-loss cost, as estimated by flux balance genome-scale models of metabolism, correlates poorly with gene evolutionary rate [11]. These prior calculations had been performed for a large number (approx. 10^4^) of randomly generated combinations of environmentally available metabolites, and using different variants of the FBA objective function (including the standard maximization of biomass production flux [14] and the minimization of metabolic adjustment upon gene deletion [21]). We started by revisiting these results, using a recently updated stoichiometric reconstruction [22,23], a different strategy for choosing a large number of environmental conditions, and independently computed evolutionary rates.

In particular, to impose environmental constraints in our FBA calculations, we generated 1,632 minimal media, each containing a nitrogen and a carbon source, in all possible combinations (see Materials and Methods for details and [24] for use of a similar strategy). Gene-loss costs were calculated across all metabolic enzyme genes and environments, using the standard FBA protocol for gene knockouts (see Material and Methods and [25]). Evolutionary rates for *S*. *cerevisiae* metabolic genes were calculated using a modified version of *d*_N_/*d*_S_ from orthologs in five related species spanning a phylogenetic timetable of roughly 10–100 million years (see Materials and Methods and [26]). Our results (Fig 2A, dark orange distribution) show that gene-loss costs weakly anticorrelate with gene evolutionary rate (Spearman’s *ρ* ranging between −0.28 and −0.03). This anticorrelation is both markedly stronger and closer to experimental results as compared to the FBA calculations in [11].

Notably, in contrast to FBA calculations previously used for this type of analysis, we limit each minimal environment to a single source of carbon and a single source of nitrogen. At the model level, such minimal media strictly enforce a kind of metabolic resource scarcity. In the absence of this scarcity, the FBA model can reroute metabolic fluxes to use alternate resources at zero cost, masking the effect of blocking individual pathways with a deletion. We also took advantage of these minimal environments to test whether or not a particular carbon or nitrogen substrate significantly influenced the anticorrelation between gene evolutionary rate and gene-loss cost. However, we do not observe that any one specific carbon or nitrogen source produces significantly stronger correlations than the other sources (*p* = 0.07, Wilcoxon rank-sum test adjusted for multiple comparisons). The strongest average anticorrelation for an individual carbon or nitrogen source is *ρ* = −0.21 for pyruvate which has a standard deviation of 0.03, compared to a mean of *ρ* = −0.19 and standard deviation of 0.04 across all combinations of carbon and nitrogen sources.

### A newly defined function-loss cost has stronger anticorrelation with evolutionary rate

Given the weakness of the correlation observed between FBA-computed gene-loss cost and gene evolutionary rate, we asked ourselves whether any step in the FBA calculation could potentially distort the estimation of the cost of gene deletion. We ended up focusing our attention on the gene-to-reaction mapping, which, in the FBA knockout calculation, translates the deletion of a gene into the corresponding flux constraints that block (potentially multiple) reactions associated with that gene (Fig 1). This mapping, expressed using simple Boolean logic, plays a particularly important role for reactions that are catalyzed by multiple enzymes (isoenzymes) or by enzyme protein complexes (Fig 1). For two isoenzymes catalyzing the same reaction, for example, deletion of one the two enzymes has no effect on the corresponding flux in a traditional FBA knockout calculation, because the other enzyme is assumed to provide full backup functionality (Fig 1B). However, abundant experimental evidence suggests that this backup effect is often limited, or condition-dependent [27–29]. The cumulative effect of this discrepancy in genome scale calculations could be quite significant, given that more than one third of the metabolic enzymes in *S*. *cerevisiae* are members of isoenzyme sets (and thus would end up incurring no cost whatsoever under standard FBA knockout calculations). We thus hypothesize that fixing this oversimplification in the assessment of gene-loss cost could have a non-negligible effect on the above-mentioned correlation estimate.

In defining a new score for the functional cost incurred upon gene deletions, we also wanted to take into account the fact that multi-functional enzymes (i.e., enzymes that catalyze more multiple distinct reactions, Fig 1C) may be under more evolutionary pressure to maintain their function(s) than genes performing only a single function, especially if all such functions are essential.

These considerations led us to define a new metric predicting the impact of gene deletions in genome-scale models. In particular, we define the *function-loss cost* of a gene as the sum of all costs incurred by removing each individual reaction catalyzed by the gene from the network (see also Materials and Methods and Fig 1), with the assumption of zero backup capacity by isoenzymes. The distribution of the newly introduced function-loss cost is substantially different from the distribution of gene-loss cost computed before (S1 and S2 Figs). Notably, for any gene that does not belong to the set of isoenzymes or to the set of multi-functional enzymes, the function-loss cost is identical to the gene-loss cost.

Interestingly, using our new function-loss cost metric as the measure of gene dispensability, we obtain a significantly stronger negative correlation between this measure and gene evolutionary rate than using gene-loss cost (Fig 2A, Wilcoxon signed-rank test *p* = 2 x 10^−240^). In fact, the mean anticorrelation between these data (*ρ* = −0.27) is even stronger than the anticorrelation observed between gene evolutionary rate and experimentally-measured gene essentialities, even though strict gene essentiality prediction accuracy obtained using function-loss cost is reduced relative to the accuracy obtained using gene-loss cost (odds ratio drops from 30 to 7). Note that the distribution of correlations between function-loss costs and evolutionary rates across different environments is similar in shape and with a similarly narrow standard deviation to the distribution previously obtained for gene-loss cost, indicating the recovery of anticorrelation obtained with the function-loss cost is not strongly dependent on nutrient choice.

### Isoenzymes play a special role in determining the anticorrelation with evolutionary rate

As a next step in our analysis, we set out to examine the contributions from isoenzymes and multi-functional enzymes to the improved negative correlations. Recalculating the correlation distributions using only the isoenzymes (Fig 2C) shows much weaker correlations between gene-loss cost and evolutionary rate than the same correlations calculated using the whole gene set (Fig 2A). For function-loss cost, restricting to isoenzymes has comparatively little effect. Conversely, for the correlation distributions excluding the isoenzymes (Fig 2B), the distribution using the gene-loss cost is significantly more negative than when using all genes in the model (Wilcoxon’s signed-rank test *p* = 3 x 10^−241^). When the correlation distributions are recalculated using only the subset of genes in the model which are multifunctional and are not isoenzymes (Fig 2D) we observe that the function-loss cost correlations are significantly weaker than gene-loss cost (Wilcoxon’s signed-rank test *p* = 1 x 10^−198^). From these observations we can conclude that it is the treatment of isoenzymes that is the cause of the stronger anticorrelation with evolutionary rate seen when using function-loss cost compared with gene-loss cost.

As a further investigation of whether the improvement gained from function-loss cost is due to a better accounting of isoenzyme deletion cost, we recomputed the impact of gene deletion in two variant “hybrid-loss cost” ways. First, we computed the gene deletion impact using the gene-loss score for all genes except the multi-functional enzyme genes, for which we use the new function-loss score (we will refer to this schema as hybrid-loss cost 1). Conversely, in a separate calculation (hybrid-loss score 2), we computed the gene deletion impact using the gene-loss score for all genes except the isoenzyme-associated genes, for which we use the new functional-loss score. We find that hybrid-loss score 2 displays a negative correlation very similar to the one observed with the full function-loss score (whereas hybrid-loss score 1 displays a negative correlation similar to gene-loss cost) (S3 Fig). Taken together with the results from Fig 2, this indicates that incorrectly accounting for the effect of isoenzyme deletion has a prominent role in the capacity to discern the relationship between the impact of gene deletion and evolutionary rate. In turn, this suggests that deletion of an isoenzyme is costly, corroborating previous arguments that true redundant functional backup is not evolutionarily sustainable [30].

In order to gather further insight into the relationship between different enzymes in an isoenzyme set, we tested the correlation between function-loss cost and evolutionary rate for different specific choices of enzymes within each set. Specifically, for each isoenzyme set, we identified the enzyme which is most conserved (slowest evolutionary rate), and the one that is least conserved (fastest evolutionary rate). Thus, across all isoenzyme pairs, we could collect a subset of all fast evolving and slow evolving isoenzymes.

**Fig 3:**
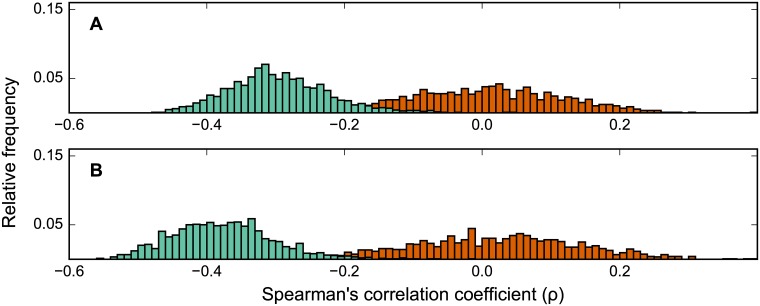
Frequency distributions of (Spearman’s rank) correlations for fast- and slow-evolving isoenzymes. Each isoenzyme in an isoenzyme gene set was categorized as one of: fast-evolving (highest evolutionary rate within the isozyme set), slow-evolving (lowest rate), or neutral. Plots show distributions of the correlation with evolutionary rate for both function-loss cost and gene-loss cost, for: (A) fast-evolving isoenzymes and (B) slow-evolving isoenzymes.

Notably, when computing the correlation between function-loss cost and evolutionary rate with the inclusion of slow-evolving isoenzymes only (Fig 3B, light green distribution), we found an average correlation of *ρ* = −0.38. This correlation is even more negative (Wilcoxon signed-rank test *p* = 1 x 10^−236^) than the mean correlation found using the whole gene set (Fig 2A, light green distribution). When similarly selecting for the fastest-evolving isoenzymes from each isoenzyme set (Fig 3A), the mean correlation of *ρ* = −0.30 is significantly less negative than that when using the slowest-evolving isoenzymes (Wilcoxon signed-rank test *p* = 4 x 10^−230^). Prior work had established that different isoenzymes catalyzing the same reaction evolve at different rates, and that this could be interpreted as a signal of subfunctionalization [31,32]. Our analysis reveals for the first time that, in computing the anticorrelation between function-loss score and evolutionary importance, excluding the fast-evolving isoenzymes results in a stronger anticorrelation than excluding the slow-evolving isoenzymes. This suggests that the historical (long-term evolutionary) importance of slow-evolving (i.e. highly conserved) genes carries more information about their experimentally measurable essentiality relative to fast-evolving counterparts.

### Modeling isoenzymes as non-redundant increases the number of predicted epistatic interactions

Given that changing how isoenzymes map to reactions in the flux balance model significantly affects the predicted cost of single gene deletions, it is interesting to ask how this change would affect the predicted cost of multiple simultaneous gene deletions. Whether the combined effect of pairs of genetic perturbations is predictable from knowledge of each individual effect constitutes a question with broad implications. In fact, deviations from simple expectations (i.e. epistasis) can significantly affect evolutionary processes [33], and can provide valuable functional information about the underlying system [34]. Previous work has investigated the capacity of FBA models to predict epistatic interactions between pairs of metabolic enzyme genes [35], also motivated by the availability of extensive experimental datasets of genetic interactions in *S*. *cerevisiae* [20]. Here, we test the ability of FBA to predict these experimentally derived interactions for two gene-to-reaction mappings. The first mapping—the default in FBA—assumes complete isoenzyme redundancy (gene-loss cost-like), while the second assumes that isoenzymes are completely non-redundant (function-loss cost-like). Our results are scored based on the ability of the model to predict the type of interaction between pairs of genes correctly: synergistic (the double knockout combines to limit cell growth more than expected from the single knockouts, e.g. synthetic lethal interactions), antagonistic (the double knockout has better cell growth than expected), or non-interacting.

**Fig 4:**
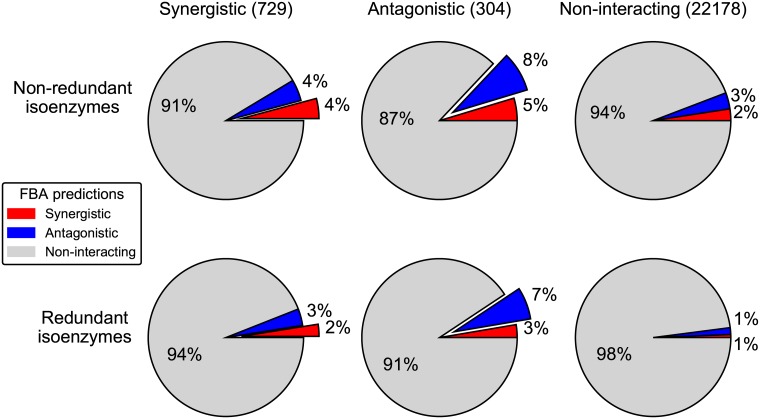
Comparison of correctly predicted epistatic interactions per gene. Each row represents one formulation of how isoenzymes map to reactions in the FBA model: either the new assumption that isoenzymes are non-redundant and so knocking out any member of the isoenzyme set knocks out the reaction (top) or the standard assumption that all isoenzymes in a set need to be knocked out in order to knock out the corresponding reaction (bottom). Columns represent experimental classification of an epistatic interaction: synergistic (left), antagonistic (middle) and non-interacting (right). The total number of these interactions are listed at the top just below the headers. The pie chart in each sextant represents the capacity of FBA to predict these interactions. The colors of the slices show the FBA model predictions (blue, red and light grey, respectively for synergistic, antagonistic and non-interacting). The offset slices show the correctly categorized genetic interactions.

Interestingly, by assuming that isoenzymes are completely non-redundant, the flux balance model correctly predicts more experimentally verified genetic interactions between pairs of genes than when assuming that isoenzymes are completely redundant (Fig 4). This increase is not limited to a particular interaction type (synergistic or antagonistic). Using either gene-to-reaction mapping assumption, the predictions are significantly better than random, for both categories of interaction (Fisher’s exact test, *p* < 0.01). The fact that changing the isoenzyme assumption increases the number of predicted epistatic interactions is perhaps unsurprising, given that, under standard gene-loss cost protocols, an isoenzyme may only be predicted to exhibit epistasis under very narrow circumstances. Namely, (1) the isoenzyme must be one of an isoenzyme pair, (2) the other deletion must be of the partner isoenzyme, and (3) the reaction they catalyze together must incur a cost penalty when blocked. However, although the sensitivity increases after changing the isoenzyme to reaction mapping, there is also a large rise in the number of false positives. Although the recall is improved, the precision is reduced (S5 Fig).

## Discussion

We have introduced function-loss cost, a new metric for quantifying the impact of the deletion of a gene based on genome-scale models of metabolism. This metric is similar to previously estimated gene-loss impacts, except for the modification of some of the basic assumptions on how the deletion of a gene translates into reaction flux constraints. The modification that ends up being responsible for recovering the expected correlation between gene deletion impact and evolutionary rate is the assumption on how isoenzyme deletion affects the corresponding reaction flux. While previous calculations assume that each enzyme in a set of isoenzymes can unconditionally perform the function in the absence of the other isoenzymes, the algorithm we use here assumes that deletion of each isoenzyme causes a complete loss of function for the cell. Based also on multiple types of analyses and observations [27,29,36], one would obviously expect the reality to be a complex combination of the above assumptions: different isoenzymes may respond differently to different environmental perturbations, and provide backup to each other to varying degrees. What our results indicate, however, is that—on average—the assumption that each isoenzyme fulfills an essential metabolic role is more consistent with the evolutionary record than the opposite assumption of isoenzymes being unconditionally, individually dispensable. On the other hand, the assumption that function-loss cost makes with respect to how the cost of multifunctional enzymes should be estimated, appears to reduce the strength of the relationship between evolutionary rate and the model predicted loss cost. The approach of taking the sum of the costs of the individual reactions was chosen to try and capture the maximum cost of a gene deletion. In the future it could be worthwhile to investigate other formulations of the cost for the loss of multifunctional enzymes, such as taking the maximum cost of the deletion of a single reaction rather than the sum of the costs of each reaction.

From the perspective of flux balance modeling, our analysis suggests that extra caution should be used when applying the classical gene-to-reaction mapping relationships to estimate the effect of gene loss, especially when using these models to understand evolutionary aspects of metabolism. As to whether our newly suggested way to deal with isoenzyme deletion will be helpful in comparisons with experimental gene deletion studies, this requires additional evaluations.

With respect to epistasis, we have shown that this modification to the assumption of how isoenzymes map to reactions results in more correctly predicted genetic interactions but also in more false positives. In prior calculations, using the standard gene-to-reaction mapping (Fig 1B), it would have been possible to detect such interactions only between two isoenzymes that are the only two catalysts for a given reaction. In any other case (e.g. an interaction involving a single isoenzyme and another arbitrary enzyme), the complete backup assumption of isoenzyme sets would completely mask any possible interaction. In the future, by integrating high throughput experimental data (such as epistasis measurements) and network structure information, it may be possible to rewrite reaction-specific gene-to-reaction relationships (using AND or OR) in order to further improve model prediction capacity.

This could prove to be a very important development for the use of constraint-based models as tools in the future study of genetics, especially in the area of biomedicine. Double gene deletions that result in cell death (synthetic lethal deletions) are an important avenue of cancer research, where the ability to induce lethality only within subpopulation of cells that carry specific mutations by inducing a perturbation to the entire population is of obvious benefit. Similarly, research into other metabolic diseases, such as fructose intolerance, could benefit from increased ability to predict unexpected changes of metabolic phenotypes caused by double gene perturbation events.

## Materials and Methods

### Yeast metabolic model and genes used in this study

This study was conducted using the Yeast 7 metabolic model of *Saccharomyces cerevisiae* metabolism [22,23], which may be obtained from http://yeast.sf.net (specifically, version 7.6). This model specifies a metabolic network consisting of 3493 reactions between 2220 metabolites, a set of 909 enzyme-encoding genes, and a set of Boolean expressions associating reactions to all possible subsets of genes that are required for catalysis (the gene-to-reaction mapping, also known as the gene-protein-reaction expression map or GPR). We identified blocked reactions in the model (reactions incapable of carrying flux) using a previously established method [37] and subsequently purged all genes associated only with these reactions from our analyses. All subsequent analyses presented in this section and the results presented in this paper were made using this subset of 792 genes. For the specific cases of correlating gene-loss cost and function-loss cost with evolutionary rate, restricting our analyses to a subset of metabolic genes did not significantly impact the outcome (S4 Fig).

### Calculation of gene evolutionary rates

The evolutionary rates of all metabolic genes included in this study were derived following the procedure described in [26]. First, *d*_N_/*d*_S_ ratios [2] (hereafter referred to simply as *k*) were obtained from [26] for each *Saccharomyces cerevisiae* model gene from its corresponding ortholog in five related yeast species: *Saccharomyces bayanus*, *Saccharomyces castellii*, *Lachancea kluyveri* (formerly *S*. *kluyveri* [38]), *Saccharomyces mikatae*, and *Saccharomyces paradoxus*. This provided, for each gene *g*, five separate strain-dependent measures of evolutionary rates ($k_{g}^{S.\;bayanus}$, $k_{g}^{S.\;castellii}$, etc.). To obtain a single representative rate for each gene ${\hat{k}}_{g}$, we first grouped all values of *k* by strain, converted these sets to rank order, and then took the average rank of each gene across these sets; that is, ${\hat{k}}_{g}=\langle \left\{{\overset{´}{k}}_{G}^{Y}\;|\;G=g\right\}\rangle$ where *ḱ*_*g*_ is the rank order of *k*_*g*_ within the set $\left\{k_{G}^{Y}\;|\;Y=y\right\}$ and *y* is the yeast comparison strain. The use of this measure of evolutionary rate allows more proteins to be analyzed than standard *d*_N_/*d*_S_, since this method does not require that a protein have orthologs in all other species used. There were 13 genes for which none of the five related species carried an appropriate ortholog. These genes were excluded from the analysis of evolutionary rates. Note that throughout the paper we refer to the averaged evolutionary rate rank score (${\hat{k}}_{g}$) as the evolutionary rate. Importantly, since all our correlations involving evolutionary rates are rank-based measures, this does not affect the outcome of these calculations.

### Prediction of gene-loss costs for *S*. *cerevisiae* metabolic genes

The gene-loss cost of each gene is calculated as the relative loss in predicted fitness of the gene-knockout mutant as compared to the predicted fitness of the wild-type yeast. Fitness predictions for the wild type and all mutants were obtained using standard flux balance analysis (FBA), which has been previously described in [14]. Briefly, FBA calculates the rate of flow (i.e. flux) of metabolites through each reaction (*v*_*i*_) in the metabolic network in such a way as to maximize the flux through a pseudo-reaction describing organism growth (*w* = *v*_biomass_), while at the same time satisfying the major constraint of steady state mass balance: $S\vec{v}=0$, where ***S*** is the stoichiometric matrix. Additional constraints may be imposed on each reaction, such that the minimum and/or maximum flux allowed through it is bounded (*α*_*i*_ ≤ *v*_*i*_ ≤ *β*_*i*_). In our FBA model we impose three such types of additional constraints on reactions: (1) reaction irreversibility constraints (*v*_*i*_ > 0), as defined by the original model; (2) constraints pertaining to environmental nutrient availability, available in S1 Table; and (3) constraints imposed by gene deletions, described here in detail. Gene deletions are translated, through the gene-to-reaction mapping, to constraints on some number of reactions (possibly zero) which limit the flux through these reactions to zero (*α*_*i*_ = 0 ≤ *v*_*i*_ ≤ *β*_*i*_ = 0). With fitness taken to be the flux through the biomass reaction, the normalized gene-loss cost of any gene *g* can be expressed as:

$$c_{g}^{\text{GLC}}=\frac{w_{\text{w}.\text{t}.}-w_{\Delta R}}{w_{\text{w}.\text{t}.}}$$

where *w*_w.t._ is the fitness of the wild type and *w*_Δ*R*_ is the fitness of the mutant with the set of reactions *R* blocked. For a reaction *r* to be in the set *R*, the gene in question (*g*) must be a necessary prerequisite for that reaction, as determined by the GPR:

R={r|GPR(r,g)=1}

### Prediction of function-loss costs for *S*. *cerevisiae* metabolic genes

The function-loss cost for each gene is calculated as the sum total of the individual costs of removing each function (reaction) the gene is responsible for from the model one-by-one, where an individual cost is represented by the fitness loss of the single-reaction knockout mutant relative to the wild type as predicted by FBA. For this purpose, a gene *g* is said to be responsible for a reaction *r* if the gene appears anywhere in that reaction’s associated GPR expression. This translates to a fairly simple adaptation of the gene-loss cost metric, which can be expressed as:

$$c_{g}^{\text{FLC}}=\underset{\left\{r\;|\text{ GPR}\left(r,g\right)\;\text{exists}\right\}}{\sum }\frac{w_{\text{w}.\text{t}.}-w_{\Delta r}}{w_{\text{w}.\text{t}.}}$$

### Generation of environmental conditions for gene-deletion impact simulations

Environmental conditions for flux balance simulations were generated by modifying a previously defined heuristic for determining minimal media that support growth [24]. First, an initial minimal medium was manually defined for the model, such that each primary nutrient (e.g. carbon and nitrogen) was provided by only a single metabolite. Our initial medium consisted of glucose, ammonium (NH_4_^+^), inorganic phosphate and sulfate, oxygen, and minerals (S1 Table). We then identified alternative carbon-providing metabolites by removing glucose from this initial medium and exhaustively testing all other metabolites for growth. Similarly, nitrogen-providing metabolites were identified by the removal of ammonium and subsequent testing of metabolites. Our final set of minimal media was constructed by taking all pair-wise combinations of carbon-providing and nitrogen-providing metabolites, together with the secondary metabolites listed previously, for which the wild-type model predicted positive growth (S1 Table).

Simulations were also conducted on several non-minimal environments representing common lab-growth media. Such so-called “rich media” were defined manually for YPD, YPLactate (both D- and L-Lactate), SD and SD−His (S1 Table). The SD-His settings were used in the epistasis investigation in order to mimic the experimental setup in [20]. Maximum import rates were restricted based on the measured uptake rate of glucose by *S*. *cerevisiae* grown in YPD where this rate is limiting.

### Calculation of epistasis

Epistatic interaction scores were calculated for each possible pair-wise interaction between genes using a standard method [39]. Epistasis (*ε*) between any pair of genes *i* and *j*, is defined as

$$\varepsilon_{ij}=w_{ij}-w_{i}\cdot w_{j}$$

where *w*_*i*_ and *w*_*j*_ represent the relative fitness of each single-gene deletion mutant and *w*_*ij*_ is the relative fitness of the double-gene deletion mutant. The relative fitness of any mutant *w*_*i*_ may be derived from the loss cost (*c*_*i*_) as *w*_i_ = 1 –*c*_i_. Predicted values of *ε* were generated using gene-loss cost and using a modified version of gene-loss cost where the ORs were replaced with ANDs for the isoenzyme gene to reaction mapping rules.

### Comparison of predicted epistasis with experimental data

In order to assess the validity of our predicted epistatic interaction scores, we compared our predictions against a data set for which these scores have been computed from experimentally observed fitness [20]. We limited our comparisons to genes for which the experimentally observed fitness of deletion mutant was no greater than two standard deviations above the fitness of the wild type, because FBA is incapable of predicting increases in fitness due to gene deletions (in the absence of other types of perturbations). We used the intermediate criteria [20] of |*ε*| > 0.08, *p* < 0.05 to classify pairs of genes in the experimental data, into synergistic interactions (negative ε), antagonistic interactions (positive ε), or non-interactions. We used a cutoff of |*ε*| > 0.0001 for the FBA predictions in Fig 4. Performance was measured by testing whether or not the epistatic classification predicted using FBA techniques matched the experimental classification.

### Data and scripts availability

All data and scripts used to generate the results presented in this work are freely available at github.com/llambourne/isoenzymes_flux_balance (doi:10.5281/zenodo.231284) and at datadryad.org (doi:10.5061/dryad.6ht2c).

## Supporting Information

S1 Fig Sampling of gene dispensability vs. evolutionary rate plots. These plots show the specific function-loss cost vs. evolutionary rate (light green dots) and gene-loss cost vs. evolutionary rate (dark orange dots) plots. The four plots compare the dot plot generated by function-loss cost and gene-loss cost by testing in the same media. The plots were selected as the most extreme media from both distributions, i.e. those which lead to the most negative and least negative Spearman’s *ρ* (top and bottom: most and least negative *ρ* for function-loss cost, respectively; middle-top and middle-bottom: most and least negative *ρ* for gene-loss).
(EPS)

S2 Fig Sampling of gene dispensability vs. evolutionary rate plots. These plots show the specific function-loss cost vs. evolutionary rate (light green dots) and gene-loss cost vs. evolutionary rate (dark orange dots) plots. The three plots show the dot plots for YPD rich media, the reference media (the default carbon/nitrogen pair from which all other media were generated) and the median *ρ*-producing media set (this is the only plot which does not compare the same environment against itself).
(EPS)

S3 Fig Hybrid-loss Costs. These plots show the frequency distributions of gene dispensability measures vs gene evolutionary rate using the hybrid-loss cost measures mentioned in the main text. The top plot matches Fig 2A (function-loss cost, light green distribution; gene-loss cost, dark orange distribution). The middle plot (indigo distribution) shows hybrid-loss cost 2, where function-loss cost is applied to isoenzymes and gene-loss cost is applied to all other genes. The bottom plot (fuchsia distribution) displays hybrid-loss cost 1, where function-loss cost is applied to multifunctional enzymes and gene-loss cost to other genes.
(EPS)

S4 Fig Blocked vs Total genes. Frequency distribution plots of gene dispensability measures vs gene evolutionary rates for all genes (B) and unblocked genes (A). Blocked genes are incapable of carrying flux under all tested media conditions.
(EPS)

S5 Fig Comparison of classification of genetic interactions. Precision/recall curves for (A) synergistic and (B) antagonistic genetic interactions using different versions of the isoenzyme to reaction mapping rules. The curves are generated by varying the ε cutoff used to define the model predictions between 0.0001 and 0.01. The ε cutoff used to define the experimental genetic interactions remains constant. Distributions of the predicted ε values using (C) the standard assumption that isoenzymes in a set are redundant and (D) the new assumption that isoenzymes in a set are non-redundant, for each possible pair of genes in the model.
(EPS)

S1 Table List of metabolites used to simulate media sets. The first row specifies the reference minimal media set that was used to generate all other minimal media sets. The next two rows, labeled “Carbons” and “Nitrogens”, list all possible carbon and nitrogen sources that could substitute for D-glucose and ammonium (NH_4_^+^) in the reference minimal media set. The final columns provide a complete listing of the metabolites within the rich media sets tested. In terms of the model: all exchange reactions not included in the above table were limited to export only (*v*_*i*_ ≥ 0). The non-carbon and non-nitrogen sources in the reference media were left as unbounded import reactions, while the carbon and nitrogen sources were limited to a *v*_*i*_ = 10.
(DOCX)
