# Supplementary material for: Upon Accounting for the Impact of Isoenzyme Loss, Gene Deletion Costs Anticorrelate with Their Evolutionary Rates
Source: PLoS One. 2017 Jan 20;12(1):e0170164. doi: 10.1371/journal.pone.0170164 (PMC5249160; doi:10.1371/journal.pone.0170164)
Supplement: S1 Table — The first row specifies the reference minimal media set that was used to generate all other minimal media sets. The next two rows, labeled “Carbons” and “Nitrogens”, list all possible carbon and nitrogen sources that could substitute for D-glucose and ammonium (NH4+) in the reference minimal media set. The final columns provide a complete listing of the metabolites within the rich media sets tested. In terms of the model: all exchange reactions not included in the above table were limited to export only (vi ≥ 0). The non-carbon and non-nitrogen sources in the reference media were left as unbounded import reactions, while the carbon and nitrogen sources were limited to a vi = 10. (DOCX) [file pone.0170164.s006.docx]

| Media Set | Metabolites |
| --- | --- |
| Reference Media Set | Glucose, ammonia, water, potassium, sodium, phosphate (Pi), sulfate, sodium, iron |
| Carbons | (1→3)-*β*-D-glucan |
|  | 2-hydroxybutyrate |
|  | 4-aminobutanoate |
|  | acetate |
|  | acetaldehyde |
|  | adenosine |
|  | 2-oxoglutarate |
|  | L-alanine |
|  | S-adenosyl-L-methionine |
|  | L-arginine |
|  | L-asparagine |
|  | L-aspartate |
|  | citrate |
|  | cytidine |
|  | ethanol |
|  | fructose |
|  | fumarate |
|  | D-galactose |
|  | D-glucosamine 6-phosphate |
|  | L-glutamine |
|  | L-glutamate |
|  | glycine |
|  | glycerol |
|  | guanosine |
|  | inosine |
|  | D-lactose |
|  | L-lactose |
|  | L-malate |
|  | maltose |
|  | D-mannose |
|  | melibiose |
|  | oxaloacetate |
|  | ornithine |
|  | adenosine 3',5'-bisphosphate |
|  | L-proline |
|  | pyruvate |
|  | D-ribose |
|  | D-sorbitol |
|  | L-serine |
|  | succinate |
|  | sucrose |
|  | L-threonine |
|  | trehalose |
|  | uridine |
|  | xanthosine |
|  | D-xylose |
|  | xylitol |
| Nitrogens | 4-aminobutanoate |
|  | adenine |
|  | adenosine |
|  | L-alanine |
|  | allantoin |
|  | allantoate |
|  | S-adenosyl-L-methionine |
|  | L-arginine |
|  | L-asparagine |
|  | L-aspartate |
|  | cytosine |
|  | cytidine |
|  | deoxycytidine |
|  | D-glucosamine 6-phosphate |
|  | L-glutamine |
|  | L-glutamate |
|  | glycine |
|  | guanosine |
|  | guanine |
|  | L-isoleucine |
|  | L-leucine |
|  | ornithine |
|  | adenosine 3',5'-bisphosphate |
|  | L-phenylalanine |
|  | L-proline |
|  | putrescine |
|  | L-serine |
|  | spermidine |
|  | spermine |
|  | L-threonine |
|  | L-tryptophan |
|  | urea |
|  | L-valine |
| YPD | oxygen, glucose, ammonia, Pi, sulfate, all 20 amino acids (L-form), potassium, sodium, biotin, choline, riboflavin, thiamine, inositol, thymidine, nicotinate, 4-aminobenzoate, (R)- pantothenate, pyridoxine uracil |
| YPLac | same as YPD, less glucose, plus D-/L-lactate |
| SD | oxygen, glucose, ammonia, Pi, sulfate, all 20 amino acids (L-form), potassium, sodium, biotin, choline, inositol, uracil |
| SD−His | same as SD, less L-histidine. |
